# Supplementary material for: Memory persistence enhancement by post-learning moderate exercise requires de novo protein synthesis in the dorsal hippocampus
Source: PLoS One. 2025 Jul 18;20(7):e0328128. doi: 10.1371/journal.pone.0328128 (PMC12273956; doi:10.1371/journal.pone.0328128)
Supplement: S1 Table — (DOCX) [file pone.0328128.s001.docx]

**Supporting information**

**S1 Table. Behavioral data from Exp. 1a for each rat**

| Rat | Condition | TDM (m) | ET-F (sec) | ET-N (sec) | TE (sec) | DR |
| --- | --- | --- | --- | --- | --- | --- |
| Experimenter 1: Learning phase | | | | | | |
| 1 | 1 hr | 19.89 | 15.58 | 28.07 | 43.65 | 0.29 |
| 2 | 1 hr | 14.00 | 23.13 | 8.92 | 32.05 | -0.44 |
| 3 | 1 hr | 20.02 | 11.65 | 17.45 | 29.10 | 0.20 |
| 4 | 1 hr | 22.37 | 27.82 | 29.79 | 57.61 | 0.03 |
| 5 | 1 hr | 22.32 | 35.59 | 21.01 | 56.60 | -0.26 |
| 6 | 1 hr | 15.57 | 50.63 | 35.18 | 85.81 | -0.18 |
| 7 | 1 hr | 25.88 | 26.69 | 31.60 | 58.29 | 0.08 |
| 8 | 1 hr | 23.88 | 26.07 | 33.95 | 60.02 | 0.13 |
| 9 | 1 hr | 19.28 | 13.09 | 15.50 | 28.59 | 0.08 |
| 1 | 24 hr | 6.85 | 1.85 | 15.43 | 17.28 | 0.79 |
| 2 | 24 hr | 15.21 | 12.70 | 17.15 | 29.85 | 0.15 |
| 3 | 24 hr | 21.51 | 26.11 | 43.07 | 69.18 | 0.25 |
| 4 | 24 hr | 12.38 | 14.45 | 5.31 | 19.76 | -0.46 |
| 5 | 24 hr | 20.86 | 28.75 | 18.77 | 47.52 | -0.21 |
| 6 | 24 hr | 21.08 | 7.30 | 17.56 | 24.86 | 0.41 |
| 7 | 24 hr | 23.87 | 12.78 | 14.83 | 27.61 | 0.07 |
| 8 | 24 hr | 16.06 | 8.38 | 10.86 | 19.24 | 0.13 |
| 9 | 24 hr | 26.60 | 21.98 | 23.24 | 45.22 | 0.03 |
| Experimenter 1: Test phase | | | | | | |
| 1 | 1 hr | 12.60 | 16.89 | 13.48 | 30.37 | -0.11 |
| 2 | 1 hr | 4.77 | 0.00 | 7.77 | 7.77 | 1.00 |
| 3 | 1 hr | 11.56 | 2.52 | 6.87 | 9.39 | 0.46 |
| 4 | 1 hr | 14.58 | 19.91 | 22.99 | 42.90 | 0.07 |
| 5 | 1 hr | 25.34 | 21.46 | 48.57 | 70.03 | 0.39 |
| 6 | 1 hr | 21.70 | 14.44 | 40.85 | 55.29 | 0.48 |
| 7 | 1 hr | 27.10 | 15.04 | 28.81 | 43.85 | 0.31 |
| 8 | 1 hr | 24.24 | 22.36 | 43.83 | 66.19 | 0.32 |
| 9 | 1 hr | 30.29 | 20.64 | 19.97 | 40.61 | -0.02 |
| 1 | 24 hr | 3.96 | 7.75 | 8.54 | 16.29 | 0.05 |
| 2 | 24 hr | 13.04 | 17.08 | 14.77 | 31.85 | -0.07 |
| 3 | 24 hr | 24.35 | 27.89 | 25.96 | 53.85 | -0.04 |
| 4 | 24 hr | 16.46 | 26.89 | 23.63 | 50.52 | -0.06 |
| 5 | 24 hr | 20.11 | 26.26 | 19.00 | 45.26 | -0.16 |
| 6 | 24 hr | 19.28 | 11.30 | 20.84 | 32.14 | 0.30 |
| 7 | 24 hr | 29.98 | 19.36 | 15.52 | 34.88 | -0.11 |
| 8 | 24 hr | 15.35 | 2.26 | 7.30 | 9.56 | 0.53 |
| 9 | 24 hr | 19.17 | 22.18 | 13.78 | 35.96 | -0.23 |
| Experimenter 2: Learning phase | | | | | | |
| 1 | 1 hr | 19.89 | 17.51 | 35.45 | 52.96 | 0.34 |
| 2 | 1 hr | 14.00 | 24.92 | 10.89 | 35.81 | -0.39 |
| 3 | 1 hr | 20.02 | 21.43 | 18.93 | 40.36 | -0.06 |
| 4 | 1 hr | 22.37 | 32.85 | 33.38 | 66.23 | 0.01 |
| 5 | 1 hr | 22.32 | 43.87 | 22.41 | 66.28 | -0.32 |
| 6 | 1 hr | 15.57 | 55.16 | 46.48 | 101.64 | -0.09 |
| 7 | 1 hr | 25.88 | 32.46 | 36.64 | 69.1 | 0.06 |
| 8 | 1 hr | 23.88 | 28.96 | 43.03 | 71.99 | 0.20 |
| 9 | 1 hr | 19.28 | 16.04 | 24.35 | 40.39 | 0.21 |
| 1 | 24 hr | 6.85 | 5.77 | 19.9 | 25.67 | 0.55 |
| 2 | 24 hr | 15.21 | 16.18 | 20.51 | 36.69 | 0.12 |
| 3 | 24 hr | 21.51 | 29.84 | 49.7 | 79.54 | 0.25 |
| 4 | 24 hr | 12.38 | 25.37 | 9.89 | 35.26 | -0.44 |
| 5 | 24 hr | 20.86 | 33.22 | 23.41 | 56.63 | -0.17 |
| 6 | 24 hr | 21.08 | 9.48 | 22.57 | 32.05 | 0.41 |
| 7 | 24 hr | 23.87 | 13.95 | 16.77 | 30.72 | 0.09 |
| 8 | 24 hr | 16.06 | 9.6 | 12.87 | 22.47 | 0.15 |
| 9 | 24 hr | 26.60 | 25.71 | 33.45 | 59.16 | 0.13 |
| Experimenter 2: Test phase | | | | | | |
| 1 | 1 hr | 12.60 | 28.78 | 19.43 | 48.21 | -0.19 |
| 2 | 1 hr | 4.77 | 0 | 8.73 | 8.73 | 1.00 |
| 3 | 1 hr | 11.56 | 3.41 | 7.31 | 10.72 | 0.36 |
| 4 | 1 hr | 14.58 | 26.71 | 24.31 | 51.02 | -0.05 |
| 5 | 1 hr | 25.34 | 28.95 | 55.38 | 84.33 | 0.31 |
| 6 | 1 hr | 21.70 | 16.96 | 48.44 | 65.4 | 0.48 |
| 7 | 1 hr | 27.10 | 23.74 | 48.11 | 71.85 | 0.34 |
| 8 | 1 hr | 24.24 | 27.56 | 52.2 | 79.76 | 0.31 |
| 9 | 1 hr | 30.29 | 23.88 | 25.42 | 49.3 | 0.03 |
| 1 | 24 hr | 3.96 | 20.2 | 12.5 | 32.7 | -0.24 |
| 2 | 24 hr | 13.04 | 18.93 | 18.31 | 37.24 | -0.02 |
| 3 | 24 hr | 24.35 | 38.55 | 31.23 | 69.78 | -0.10 |
| 4 | 24 hr | 16.46 | 28.06 | 33.76 | 61.82 | 0.09 |
| 5 | 24 hr | 20.11 | 33.47 | 22.27 | 55.74 | -0.20 |
| 6 | 24 hr | 19.28 | 15.11 | 25.06 | 40.17 | 0.25 |
| 7 | 24 hr | 29.98 | 22.76 | 18.81 | 41.57 | -0.10 |
| 8 | 24 hr | 15.35 | 2.5 | 7.93 | 10.43 | 0.52 |
| 9 | 24 hr | 19.17 | 29.73 | 18.76 | 48.49 | -0.23 |

TDM: total distance moved; ET-F: exploration time of familiar (F) location object; ET-N: exploration time of familiar (N) location object; TE: total (F+N) object exploration time; DR: discrimination ratio.
