# Supplementary material for: Memory persistence enhancement by post-learning moderate exercise requires de novo protein synthesis in the dorsal hippocampus
Source: PLoS One. 2025 Jul 18;20(7):e0328128. doi: 10.1371/journal.pone.0328128 (PMC12273956; doi:10.1371/journal.pone.0328128)
Supplement: S2 Table — (DOCX) [file pone.0328128.s002.docx]

**Supporting information**

**S2 Table. Behavioral data from Exp. 2 for each rat**

| Rat | Condition | TDM (m) | ET-F (sec) | ET-N (sec) | TE (sec) | DR |
| --- | --- | --- | --- | --- | --- | --- |
| Learning phase | |  |  |  |  |  |
| 1 | Sed | 21.12 | 58.04 | 30.77 | 88.81 | -0.31 |
| 2 | Sed | 16.33 | 23.70 | 21.82 | 45.52 | -0.04 |
| 3 | Sed | 15.67 | 22.74 | 32.49 | 55.23 | 0.18 |
| 4 | Sed | 7.18 | 16.61 | 17.26 | 33.87 | 0.02 |
| 5 | Sed | 16.45 | 46.42 | 42.10 | 88.52 | -0.05 |
| 6 | Sed | 27.74 | 19.75 | 44.11 | 63.86 | 0.38 |
| 7 | Sed | 5.02 | 11.34 | 18.12 | 29.46 | 0.23 |
| 8 | Sed | 25.06 | 34.47 | 24.75 | 59.22 | -0.16 |
| 9 | Sed | 16.71 | 16.34 | 22.93 | 39.27 | 0.17 |
| 10 | Sed | 23.35 | 40.87 | 24.91 | 65.78 | -0.24 |
| 11 | Sed | 19.27 | 31.92 | 21.82 | 53.74 | -0.19 |
| 12 | Sed | 11.71 | 16.87 | 9.54 | 26.41 | -0.28 |
| 13 | Sed | 6.06 | 10.59 | 11.68 | 22.27 | 0.05 |
| 14 | Sed | 17.10 | 11.85 | 21.49 | 33.34 | 0.29 |
| 15 | Sed | 16.27 | 20.72 | 16.30 | 37.02 | -0.12 |
| 16 | Sed | 21.36 | 18.57 | 27.10 | 45.67 | 0.19 |
| 17 | Sed | 12.30 | 26.16 | 24.92 | 51.08 | -0.02 |
| 18 | Sed | 18.23 | 32.79 | 38.75 | 71.54 | 0.08 |
| 1 | AME | 21.60 | 36.22 | 44.17 | 80.39 | 0.10 |
| 2 | AME | 25.15 | 32.53 | 30.66 | 63.19 | -0.03 |
| 3 | AME | 7.76 | 4.70 | 24.27 | 28.97 | 0.68 |
| 4 | AME | 3.60 | 0.00 | 38.72 | 38.72 | 1.00 |
| 5 | AME | 20.58 | 45.05 | 40.19 | 85.24 | -0.06 |
| 6 | AME | 17.20 | 44.06 | 47.03 | 91.09 | 0.03 |
| 7 | AME | 10.25 | 31.92 | 11.75 | 43.67 | -0.46 |
| 8 | AME | 26.10 | 12.34 | 22.87 | 35.21 | 0.30 |
| 9 | AME | 13.32 | 3.67 | 37.97 | 41.64 | 0.82 |
| 10 | AME | 20.99 | 42.92 | 39.62 | 82.54 | -0.04 |
| 11 | AME | 22.62 | 27.24 | 31.11 | 58.35 | 0.07 |
| 12 | AME | 15.60 | 22.90 | 10.66 | 33.56 | -0.36 |
| 13 | AME | 13.60 | 31.65 | 34.13 | 65.78 | 0.04 |
| 14 | AME | 14.74 | 46.43 | 24.25 | 70.68 | -0.31 |
| 15 | AME | 10.56 | 2.59 | 6.79 | 9.38 | 0.45 |
| 16 | AME | 10.69 | 18.44 | 6.26 | 24.70 | -0.49 |
| 17 | AME | 15.57 | 21.68 | 28.42 | 50.10 | 0.13 |
| 18 | AME | 22.92 | 17.14 | 24.27 | 41.41 | 0.17 |
| Test phase | | |  |  |  |  |
| 1 | Sed | 14.69 | 29.75 | 15.75 | 45.50 | -0.31 |
| 2 | Sed | 24.34 | 29.89 | 34.67 | 64.56 | 0.07 |
| 3 | Sed | 19.42 | 35.76 | 25.67 | 61.43 | -0.16 |
| 4 | Sed | 7.70 | 7.69 | 33.86 | 41.55 | 0.63 |
| 5 | Sed | 20.41 | 17.04 | 37.37 | 54.41 | 0.37 |
| 6 | Sed | 25.85 | 47.95 | 38.02 | 85.97 | -0.12 |
| 7 | Sed | 7.68 | 22.37 | 13.97 | 36.34 | -0.23 |
| 8 | Sed | 22.46 | 21.32 | 27.18 | 48.50 | 0.12 |
| 9 | Sed | 18.27 | 30.37 | 21.48 | 51.85 | -0.17 |
| 10 | Sed | 23.58 | 18.59 | 38.22 | 56.81 | 0.35 |
| 11 | Sed | 29.40 | 24.20 | 19.41 | 43.61 | -0.11 |
| 12 | Sed | 12.58 | 10.85 | 17.80 | 28.65 | 0.24 |
| 13 | Sed | 12.23 | 12.39 | 15.52 | 27.91 | 0.11 |
| 14 | Sed | 16.89 | 26.44 | 24.09 | 50.53 | -0.05 |
| 15 | Sed | 11.19 | 7.62 | 15.98 | 23.60 | 0.35 |
| 16 | Sed | 10.49 | 6.71 | 18.18 | 24.89 | 0.46 |
| 17 | Sed | 15.90 | 23.49 | 23.90 | 47.39 | 0.01 |
| 18 | Sed | 18.77 | 36.30 | 43.44 | 79.74 | 0.09 |
| 1 | AME | 23.79 | 15.23 | 33.59 | 48.82 | 0.38 |
| 2 | AME | 13.93 | 24.93 | 31.63 | 56.56 | 0.12 |
| 3 | AME | 15.06 | 21.75 | 27.08 | 48.83 | 0.11 |
| 4 | AME | 6.05 | 10.84 | 33.65 | 44.49 | 0.51 |
| 5 | AME | 21.24 | 31.15 | 32.24 | 63.39 | 0.02 |
| 6 | AME | 20.71 | 34.89 | 46.00 | 80.89 | 0.14 |
| 7 | AME | 15.74 | 8.91 | 14.08 | 22.99 | 0.22 |
| 8 | AME | 27.09 | 33.37 | 16.34 | 49.71 | -0.34 |
| 9 | AME | 15.86 | 24.85 | 7.07 | 31.92 | -0.56 |
| 10 | AME | 17.68 | 25.07 | 31.21 | 56.28 | 0.11 |
| 11 | AME | 24.25 | 32.11 | 34.64 | 66.75 | 0.04 |
| 12 | AME | 6.85 | 4.03 | 18.31 | 22.34 | 0.64 |
| 13 | AME | 9.47 | 26.65 | 22.49 | 49.14 | -0.08 |
| 14 | AME | 12.19 | 13.41 | 26.15 | 39.56 | 0.32 |
| 15 | AME | 10.35 | 5.26 | 26.52 | 31.78 | 0.67 |
| 16 | AME | 9.66 | 14.46 | 21.16 | 35.62 | 0.19 |
| 17 | AME | 12.36 | 5.09 | 14.74 | 19.83 | 0.49 |
| 18 | AME | 21.92 | 18.43 | 27.88 | 46.31 | 0.20 |

Sed: sedentary control; AME: acute moderate exercise; TDM: total distance moved; ET-F: exploration time of familiar (F) location object; ET-N: exploration time of familiar (N) location object; TE: total (F+N) object exploration time; DR: discrimination ratio.
