# Supplementary material for: Memory persistence enhancement by post-learning moderate exercise requires de novo protein synthesis in the dorsal hippocampus
Source: PLoS One. 2025 Jul 18;20(7):e0328128. doi: 10.1371/journal.pone.0328128 (PMC12273956; doi:10.1371/journal.pone.0328128)
Supplement: S3 Table — (DOCX) [file pone.0328128.s003.docx]

**Supporting information**

**S3 Table. Behavioral data from Exp. 3 for each rat**

| Rat | Exercise | Drug | TDM (m) | ET-F (sec) | ET-N (sec) | TE (sec) | DR |
| --- | --- | --- | --- | --- | --- | --- | --- |
| Learning phase | | |  |  |  |  |  |
| 1 | Sed | Sal | 26.08 | 40.67 | 31.86 | 72.53 | -0.12 |
| 2 | Sed | Sal | 17.31 | 26.21 | 21.29 | 47.50 | -0.10 |
| 3 | Sed | Sal | 31.03 | 36.32 | 35.98 | 72.30 | 0.00 |
| 4 | Sed | Sal | 16.20 | 15.14 | 28.52 | 43.66 | 0.31 |
| 5 | Sed | Sal | 20.12 | 20.19 | 33.05 | 53.24 | 0.24 |
| 6 | Sed | Sal | 29.27 | 35.25 | 15.25 | 50.50 | -0.40 |
| 7 | Sed | Sal | 19.07 | 27.01 | 15.77 | 42.78 | -0.26 |
| 8 | Sed | Sal | 17.57 | 40.54 | 24.22 | 64.76 | -0.25 |
| 9 | Sed | Sal | 30.71 | 38.66 | 26.45 | 65.11 | -0.19 |
| 10 | Sed | Sal | 18.22 | 22.45 | 32.55 | 55.00 | 0.18 |
| 11 | Sed | Sal | 23.32 | 20.56 | 30.04 | 50.60 | 0.19 |
| 12 | Sed | Sal | 22.92 | 19.66 | 20.64 | 40.30 | 0.02 |
| 13 | Sed | Sal | 28.75 | 25.65 | 41.42 | 67.07 | 0.24 |
| 14 | Sed | Sal | 30.37 | 31.05 | 35.89 | 66.94 | 0.07 |
| 15 | Sed | Sal | 20.78 | 31.00 | 32.41 | 63.41 | 0.02 |
| 16 | Sed | Sal | 21.30 | 12.68 | 11.08 | 23.76 | -0.07 |
| 17 | Sed | Sal | 14.02 | 12.94 | 23.94 | 36.88 | 0.30 |
| 18 | Sed | Sal | 23.78 | 52.13 | 32.04 | 84.17 | -0.24 |
| 19 | Sed | Sal | 26.31 | 16.54 | 15.17 | 31.71 | -0.04 |
| 20 | AME | Sal | 15.18 | 20.25 | 11.25 | 31.50 | -0.29 |
| 21 | AME | Sal | 25.43 | 21.62 | 16.06 | 37.68 | -0.15 |
| 22 | AME | Sal | 7.31 | 11.59 | 42.56 | 54.15 | 0.57 |
| 23 | AME | Sal | 13.16 | 9.98 | 19.97 | 29.95 | 0.33 |
| 24 | AME | Sal | 23.97 | 45.43 | 31.52 | 76.95 | -0.18 |
| 25 | AME | Sal | 23.02 | 37.13 | 25.91 | 63.04 | -0.18 |
| 26 | AME | Sal | 25.74 | 28.14 | 13.01 | 41.15 | -0.37 |
| 27 | AME | Sal | 18.43 | 16.85 | 21.88 | 38.73 | 0.13 |
| 28 | AME | Sal | 21.21 | 27.02 | 12.20 | 39.22 | -0.38 |
| 29 | AME | Sal | 28.70 | 27.10 | 25.94 | 53.04 | -0.02 |
| 30 | AME | Sal | 16.97 | 8.02 | 17.18 | 25.20 | 0.36 |
| 31 | AME | Sal | 14.83 | 14.80 | 11.55 | 26.35 | -0.12 |
| 32 | AME | Sal | 16.98 | 15.99 | 30.28 | 46.27 | 0.31 |
| 33 | AME | Sal | 25.60 | 20.66 | 22.56 | 43.22 | 0.04 |
| 34 | AME | Sal | 24.48 | 46.40 | 18.38 | 64.78 | -0.43 |
| 35 | AME | Sal | 25.78 | 20.23 | 37.20 | 57.43 | 0.30 |
| 36 | AME | Sal | 7.21 | 0.00 | 15.21 | 15.21 | 1.00 |
| 37 | AME | Sal | 23.66 | 25.06 | 21.56 | 46.62 | -0.08 |
| 1 | Sed | ANI | 26.51 | 34.41 | 28.13 | 62.54 | -0.10 |
| 2 | Sed | ANI | 17.36 | 14.63 | 14.92 | 29.55 | 0.01 |
| 3 | Sed | ANI | 24.35 | 54.80 | 24.08 | 78.88 | -0.39 |
| 4 | Sed | ANI | 14.23 | 38.64 | 8.05 | 46.69 | -0.66 |
| 5 | Sed | ANI | 23.91 | 23.14 | 38.28 | 61.42 | 0.25 |
| 6 | Sed | ANI | 23.51 | 8.98 | 13.13 | 22.11 | 0.19 |
| 7 | Sed | ANI | 22.45 | 32.64 | 34.13 | 66.77 | 0.02 |
| 8 | Sed | ANI | 20.95 | 30.45 | 16.55 | 47.00 | -0.30 |
| 9 | Sed | ANI | 27.67 | 40.21 | 39.98 | 80.19 | 0.00 |
| 10 | Sed | ANI | 13.66 | 30.69 | 18.45 | 49.14 | -0.25 |
| 11 | Sed | ANI | 22.02 | 24.53 | 32.56 | 57.09 | 0.14 |
| 12 | Sed | ANI | 24.60 | 28.77 | 23.26 | 52.03 | -0.11 |
| 13 | Sed | ANI | 18.64 | 17.59 | 21.83 | 39.42 | 0.11 |
| 14 | Sed | ANI | 26.78 | 24.39 | 18.51 | 42.90 | -0.14 |
| 15 | Sed | ANI | 24.40 | 8.70 | 26.41 | 35.11 | 0.50 |
| 16 | Sed | ANI | 19.92 | 13.77 | 28.79 | 42.56 | 0.35 |
| 17 | Sed | ANI | 16.73 | 13.83 | 19.37 | 33.20 | 0.17 |
| 18 | Sed | ANI | 30.51 | 47.05 | 55.72 | 102.80 | 0.08 |
| 19 | Sed | ANI | 24.22 | 34.02 | 30.12 | 64.14 | -0.06 |
| 20 | AME | ANI | 18.56 | 12.55 | 31.79 | 44.34 | 0.43 |
| 21 | AME | ANI | 23.15 | 24.00 | 31.58 | 55.58 | 0.14 |
| 22 | AME | ANI | 26.49 | 39.20 | 18.97 | 58.17 | -0.35 |
| 23 | AME | ANI | 23.21 | 23.76 | 14.59 | 38.35 | -0.24 |
| 24 | AME | ANI | 23.84 | 13.07 | 21.56 | 34.63 | 0.25 |
| 25 | AME | ANI | 26.80 | 23.84 | 26.01 | 49.85 | 0.04 |
| 26 | AME | ANI | 22.29 | 14.75 | 24.21 | 38.96 | 0.24 |
| 27 | AME | ANI | 19.09 | 15.12 | 18.74 | 33.86 | 0.11 |
| 28 | AME | ANI | 20.58 | 25.51 | 43.60 | 69.11 | 0.26 |
| 29 | AME | ANI | 28.03 | 27.11 | 22.66 | 49.77 | -0.09 |
| 30 | AME | ANI | 14.25 | 35.19 | 6.63 | 41.82 | -0.68 |
| 31 | AME | ANI | 13.14 | 12.77 | 12.39 | 25.16 | -0.02 |
| 32 | AME | ANI | 19.84 | 16.79 | 23.37 | 40.16 | 0.16 |
| 33 | AME | ANI | 33.87 | 18.52 | 25.94 | 44.46 | 0.17 |
| 34 | AME | ANI | 30.16 | 23.90 | 43.24 | 67.14 | 0.29 |
| 35 | AME | ANI | 27.40 | 37.24 | 42.28 | 79.52 | 0.06 |
| 36 | AME | ANI | 24.03 | 30.39 | 34.53 | 64.92 | 0.06 |
| 37 | AME | ANI | 23.25 | 33.04 | 29.80 | 62.84 | -0.05 |
| Test phase | | |  |  |  |  |  |
| 1 | Sed | Sal | 26.01 | 22.02 | 30.91 | 52.93 | 0.17 |
| 2 | Sed | Sal | 14.15 | 16.62 | 19.92 | 36.54 | 0.09 |
| 3 | Sed | Sal | 29.31 | 23.03 | 18.05 | 41.08 | -0.12 |
| 4 | Sed | Sal | 13.15 | 10.04 | 10.74 | 20.78 | 0.03 |
| 5 | Sed | Sal | 20.36 | 12.31 | 6.92 | 19.23 | -0.28 |
| 6 | Sed | Sal | 25.28 | 11.85 | 11.92 | 23.77 | 0.00 |
| 7 | Sed | Sal | 18.53 | 26.30 | 33.89 | 60.19 | 0.13 |
| 8 | Sed | Sal | 16.03 | 33.04 | 23.49 | 56.53 | -0.17 |
| 9 | Sed | Sal | 27.82 | 25.36 | 27.40 | 52.76 | 0.04 |
| 10 | Sed | Sal | 18.81 | 18.57 | 25.42 | 43.99 | 0.16 |
| 11 | Sed | Sal | 22.66 | 12.29 | 23.73 | 36.02 | 0.32 |
| 12 | Sed | Sal | 11.04 | 12.70 | 7.25 | 19.95 | -0.27 |
| 13 | Sed | Sal | 19.26 | 25.05 | 17.55 | 42.60 | -0.18 |
| 14 | Sed | Sal | 23.19 | 33.37 | 16.67 | 50.04 | -0.33 |
| 15 | Sed | Sal | 15.00 | 19.43 | 31.42 | 50.85 | 0.24 |
| 16 | Sed | Sal | 23.02 | 15.77 | 14.71 | 30.48 | -0.03 |
| 17 | Sed | Sal | 16.35 | 10.51 | 17.29 | 27.80 | 0.24 |
| 18 | Sed | Sal | 26.56 | 32.35 | 27.87 | 60.22 | -0.07 |
| 19 | Sed | Sal | 21.88 | 11.59 | 21.66 | 33.25 | 0.30 |
| 20 | AME | Sal | 12.36 | 10.94 | 24.67 | 35.61 | 0.39 |
| 21 | AME | Sal | 24.45 | 4.61 | 21.44 | 26.05 | 0.65 |
| 22 | AME | Sal | 5.78 | 5.80 | 14.66 | 20.46 | 0.43 |
| 23 | AME | Sal | 18.48 | 11.26 | 28.17 | 39.43 | 0.43 |
| 24 | AME | Sal | 25.31 | 32.42 | 27.02 | 59.44 | -0.09 |
| 25 | AME | Sal | 27.21 | 22.53 | 28.60 | 51.13 | 0.12 |
| 26 | AME | Sal | 15.06 | 14.17 | 28.24 | 42.41 | 0.33 |
| 27 | AME | Sal | 10.56 | 11.39 | 7.45 | 18.84 | -0.21 |
| 28 | AME | Sal | 17.15 | 6.13 | 17.84 | 23.97 | 0.49 |
| 29 | AME | Sal | 19.08 | 12.16 | 11.55 | 23.71 | -0.03 |
| 30 | AME | Sal | 10.57 | 5.29 | 16.23 | 21.52 | 0.51 |
| 31 | AME | Sal | 17.01 | 12.04 | 24.75 | 36.79 | 0.35 |
| 32 | AME | Sal | 18.11 | 8.93 | 37.54 | 46.47 | 0.62 |
| 33 | AME | Sal | 32.55 | 26.62 | 17.94 | 44.56 | -0.19 |
| 34 | AME | Sal | 33.41 | 28.58 | 28.76 | 57.34 | 0.00 |
| 35 | AME | Sal | 23.21 | 15.33 | 42.66 | 57.99 | 0.47 |
| 36 | AME | Sal | 18.47 | 20.56 | 24.14 | 44.70 | 0.08 |
| 37 | AME | Sal | 17.19 | 11.19 | 19.93 | 31.12 | 0.28 |
| 1 | Sed | ANI | 23.96 | 31.36 | 23.95 | 55.31 | -0.13 |
| 2 | Sed | ANI | 15.70 | 5.72 | 23.16 | 28.88 | 0.60 |
| 3 | Sed | ANI | 27.03 | 36.05 | 76.03 | 112.10 | 0.36 |
| 4 | Sed | ANI | 20.08 | 23.18 | 10.98 | 34.16 | -0.36 |
| 5 | Sed | ANI | 25.03 | 25.89 | 38.07 | 63.96 | 0.19 |
| 6 | Sed | ANI | 27.63 | 11.23 | 15.15 | 26.38 | 0.15 |
| 7 | Sed | ANI | 11.04 | 8.21 | 14.08 | 22.29 | 0.26 |
| 8 | Sed | ANI | 19.11 | 27.99 | 13.49 | 41.48 | -0.35 |
| 9 | Sed | ANI | 28.63 | 38.65 | 22.40 | 61.05 | -0.27 |
| 10 | Sed | ANI | 16.19 | 33.50 | 24.32 | 57.82 | -0.16 |
| 11 | Sed | ANI | 23.15 | 20.84 | 33.77 | 54.61 | 0.24 |
| 12 | Sed | ANI | 22.47 | 16.11 | 13.08 | 29.19 | -0.10 |
| 13 | Sed | ANI | 22.02 | 15.67 | 17.61 | 33.28 | 0.06 |
| 14 | Sed | ANI | 26.98 | 26.31 | 17.55 | 43.86 | -0.20 |
| 15 | Sed | ANI | 17.90 | 4.73 | 13.65 | 18.38 | 0.49 |
| 16 | Sed | ANI | 25.37 | 7.84 | 20.52 | 28.36 | 0.45 |
| 17 | Sed | ANI | 15.29 | 15.37 | 13.01 | 28.38 | -0.08 |
| 18 | Sed | ANI | 27.56 | 42.80 | 36.58 | 79.38 | -0.08 |
| 19 | Sed | ANI | 26.01 | 29.96 | 30.08 | 60.04 | 0.00 |
| 20 | AME | ANI | 23.99 | 16.37 | 29.65 | 46.02 | 0.29 |
| 21 | AME | ANI | 17.43 | 11.24 | 18.31 | 29.55 | 0.24 |
| 22 | AME | ANI | 13.55 | 38.90 | 29.16 | 68.06 | -0.14 |
| 23 | AME | ANI | 26.10 | 21.03 | 18.04 | 39.07 | -0.08 |
| 24 | AME | ANI | 24.36 | 26.66 | 19.77 | 46.43 | -0.15 |
| 25 | AME | ANI | 28.28 | 15.03 | 15.77 | 30.80 | 0.02 |
| 26 | AME | ANI | 17.59 | 19.78 | 12.30 | 32.08 | -0.23 |
| 27 | AME | ANI | 12.73 | 15.96 | 17.39 | 33.35 | 0.04 |
| 28 | AME | ANI | 22.85 | 19.61 | 24.78 | 44.39 | 0.12 |
| 29 | AME | ANI | 21.78 | 16.82 | 13.20 | 30.02 | -0.12 |
| 30 | AME | ANI | 21.20 | 17.88 | 25.87 | 43.75 | 0.18 |
| 31 | AME | ANI | 22.52 | 16.80 | 20.13 | 36.93 | 0.09 |
| 32 | AME | ANI | 18.64 | 16.58 | 21.74 | 38.32 | 0.13 |
| 33 | AME | ANI | 34.37 | 29.16 | 36.56 | 65.72 | 0.11 |
| 34 | AME | ANI | 30.42 | 50.47 | 28.02 | 78.49 | -0.29 |
| 35 | AME | ANI | 25.11 | 26.68 | 31.36 | 58.04 | 0.08 |
| 36 | AME | ANI | 18.86 | 30.14 | 8.87 | 39.01 | -0.55 |
| 37 | AME | ANI | 30.75 | 26.13 | 24.13 | 50.26 | -0.04 |

Sed: sedentary control; AME: acute moderate exercise; Sal: saline; ANI: anisomycin; TDM: total distance moved; ET-F: exploration time of familiar (F) location object; ET-N: exploration time of familiar (N) location object; TE: total (F+N) object exploration time; DR: discrimination ratio.
