# Supplementary material for: Memory persistence enhancement by post-learning moderate exercise requires de novo protein synthesis in the dorsal hippocampus
Source: PLoS One. 2025 Jul 18;20(7):e0328128. doi: 10.1371/journal.pone.0328128 (PMC12273956; doi:10.1371/journal.pone.0328128)
Supplement: S4 Table — (DOCX) [file pone.0328128.s004.docx]

**Supporting information**

**S4 Table. Behavioral data from Exp. 4 for each rat**

| Rat | Exercise | Drug | TDM (m) | ET-F (sec) | ET-N (sec) | TE (sec) | DR |
| --- | --- | --- | --- | --- | --- | --- | --- |
| Learning phase | | |  |  |  |  |  |
| 1 | Sed | Sal | 19.60 | 11.80 | 40.26 | 52.06 | 0.55 |
| 2 | Sed | Sal | 18.79 | 20.73 | 20.86 | 41.59 | 0.00 |
| 3 | Sed | Sal | 25.15 | 28.43 | 19.94 | 48.37 | -0.18 |
| 4 | Sed | Sal | 14.14 | 42.11 | 18.83 | 60.94 | -0.38 |
| 5 | Sed | Sal | 24.73 | 44.91 | 21.24 | 66.15 | -0.36 |
| 6 | Sed | Sal | 26.39 | 30.74 | 34.67 | 65.41 | 0.06 |
| 7 | Sed | Sal | 21.33 | 27.02 | 33.72 | 60.74 | 0.11 |
| 8 | Sed | Sal | 28.08 | 39.59 | 41.64 | 81.23 | 0.03 |
| 9 | Sed | Sal | 10.56 | 8.86 | 27.75 | 36.61 | 0.52 |
| 10 | Sed | Sal | 14.76 | 29.13 | 39.84 | 68.97 | 0.16 |
| 11 | Sed | Sal | 21.83 | 14.17 | 28.20 | 42.37 | 0.33 |
| 12 | Sed | Sal | 21.15 | 69.50 | 29.22 | 98.72 | -0.41 |
| 13 | Sed | Sal | 27.17 | 25.62 | 26.09 | 51.71 | 0.01 |
| 14 | Sed | Sal | 25.54 | 49.47 | 30.65 | 80.12 | -0.23 |
| 15 | Sed | Sal | 38.76 | 30.33 | 38.71 | 69.04 | 0.12 |
| 16 | AME | Sal | 22.32 | 20.17 | 50.56 | 70.73 | 0.43 |
| 17 | AME | Sal | 30.02 | 8.48 | 24.65 | 33.13 | 0.49 |
| 18 | AME | Sal | 14.76 | 13.31 | 18.61 | 31.92 | 0.17 |
| 19 | AME | Sal | 13.79 | 11.89 | 25.09 | 36.98 | 0.36 |
| 20 | AME | Sal | 16.16 | 21.17 | 14.10 | 35.27 | -0.20 |
| 21 | AME | Sal | 14.85 | 20.06 | 34.73 | 54.79 | 0.27 |
| 22 | AME | Sal | 21.05 | 27.73 | 26.75 | 54.48 | -0.02 |
| 23 | AME | Sal | 20.46 | 36.67 | 19.62 | 56.29 | -0.30 |
| 24 | AME | Sal | 19.35 | 10.51 | 31.97 | 42.48 | 0.51 |
| 25 | AME | Sal | 26.09 | 19.08 | 19.54 | 38.62 | 0.01 |
| 26 | AME | Sal | 29.01 | 33.01 | 24.91 | 57.92 | -0.14 |
| 27 | AME | Sal | 10.17 | 24.95 | 33.80 | 58.75 | 0.15 |
| 28 | AME | Sal | 34.37 | 22.04 | 30.53 | 52.57 | 0.16 |
| 29 | AME | Sal | 21.52 | 24.35 | 22.40 | 46.75 | -0.04 |
| 1 | Sed | ANI | 21.48 | 29.8 | 29.66 | 59.46 | 0.00 |
| 2 | Sed | ANI | 20.02 | 25.37 | 25.08 | 50.45 | -0.01 |
| 3 | Sed | ANI | 27.21 | 18.37 | 21.38 | 39.75 | 0.08 |
| 4 | Sed | ANI | 19.32 | 23.87 | 30.7 | 54.57 | 0.13 |
| 5 | Sed | ANI | 19.00 | 9.94 | 16.94 | 26.88 | 0.26 |
| 6 | Sed | ANI | 27.75 | 39.09 | 18.56 | 57.65 | -0.36 |
| 7 | Sed | ANI | 23.40 | 25.34 | 30.83 | 56.17 | 0.10 |
| 8 | Sed | ANI | 25.52 | 26.89 | 35.6 | 62.49 | 0.14 |
| 9 | Sed | ANI | 14.74 | 25.18 | 25.62 | 50.80 | 0.01 |
| 10 | Sed | ANI | 19.88 | 30.4 | 28.81 | 59.21 | -0.03 |
| 11 | Sed | ANI | 21.22 | 18.76 | 9.39 | 28.15 | -0.33 |
| 12 | Sed | ANI | 24.48 | 31.5 | 33.68 | 65.18 | 0.03 |
| 13 | Sed | ANI | 21.55 | 22.57 | 14.31 | 36.88 | -0.22 |
| 14 | Sed | ANI | 34.33 | 32.74 | 40.11 | 72.85 | 0.10 |
| 15 | Sed | ANI | 30.51 | 44.78 | 23.88 | 68.66 | -0.30 |
| 16 | AME | ANI | 24.12 | 51.12 | 44.55 | 95.67 | -0.07 |
| 17 | AME | ANI | 31.57 | 23.88 | 19.36 | 43.24 | -0.10 |
| 18 | AME | ANI | 8.85 | 4.31 | 16.13 | 20.44 | 0.58 |
| 19 | AME | ANI | 24.65 | 36.62 | 21.44 | 58.06 | -0.26 |
| 20 | AME | ANI | 22.49 | 31.15 | 28.76 | 59.91 | -0.04 |
| 21 | AME | ANI | 6.17 | 0.00 | 30.25 | 30.25 | 1.00 |
| 22 | AME | ANI | 25.74 | 47.59 | 39.98 | 87.57 | -0.09 |
| 23 | AME | ANI | 22.71 | 12.44 | 24.80 | 37.24 | 0.33 |
| 24 | AME | ANI | 20.79 | 28.66 | 19.52 | 48.18 | -0.19 |
| 25 | AME | ANI | 18.38 | 13.42 | 7.56 | 20.98 | -0.28 |
| 26 | AME | ANI | 29.23 | 30.13 | 32.89 | 63.02 | 0.04 |
| 27 | AME | ANI | 6.34 | 39.82 | 15.18 | 55.00 | -0.45 |
| 28 | AME | ANI | 28.36 | 25.34 | 28.95 | 54.29 | 0.07 |
| 29 | AME | ANI | 22.33 | 31.30 | 44.20 | 75.50 | 0.17 |
| Test phase | | |  |  |  |  |  |
| 1 | Sed | Sal | 19.08 | 31.41 | 23.38 | 54.79 | -0.15 |
| 2 | Sed | Sal | 17.53 | 10.19 | 18.81 | 29.00 | 0.30 |
| 3 | Sed | Sal | 18.80 | 32.88 | 45.19 | 78.07 | 0.16 |
| 4 | Sed | Sal | 14.49 | 29.88 | 24.27 | 54.15 | -0.10 |
| 5 | Sed | Sal | 25.82 | 29.77 | 36.50 | 66.27 | 0.10 |
| 6 | Sed | Sal | 30.92 | 13.57 | 33.52 | 47.09 | 0.42 |
| 7 | Sed | Sal | 19.52 | 36.68 | 22.23 | 58.91 | -0.25 |
| 8 | Sed | Sal | 28.73 | 38.16 | 45.93 | 84.09 | 0.09 |
| 9 | Sed | Sal | 21.21 | 36.96 | 27.59 | 64.55 | -0.15 |
| 10 | Sed | Sal | 17.94 | 19.47 | 22.83 | 42.30 | 0.08 |
| 11 | Sed | Sal | 19.02 | 7.49 | 18.86 | 26.35 | 0.43 |
| 12 | Sed | Sal | 22.02 | 37.97 | 25.57 | 63.54 | -0.20 |
| 13 | Sed | Sal | 29.63 | 12.95 | 23.09 | 36.04 | 0.28 |
| 14 | Sed | Sal | 31.17 | 29.80 | 38.10 | 67.90 | 0.12 |
| 15 | Sed | Sal | 39.94 | 31.51 | 41.57 | 73.08 | 0.14 |
| 16 | AME | Sal | 20.80 | 40.99 | 35.92 | 76.91 | -0.07 |
| 17 | AME | Sal | 26.06 | 32.65 | 23.21 | 55.86 | -0.17 |
| 18 | AME | Sal | 9.32 | 22.94 | 16.22 | 39.16 | -0.17 |
| 19 | AME | Sal | 20.84 | 11.44 | 27.76 | 39.20 | 0.42 |
| 20 | AME | Sal | 12.12 | 6.94 | 11.51 | 18.45 | 0.25 |
| 21 | AME | Sal | 9.89 | 9.67 | 14.54 | 24.21 | 0.20 |
| 22 | AME | Sal | 23.61 | 32.15 | 37.46 | 69.61 | 0.08 |
| 23 | AME | Sal | 20.95 | 22.08 | 29.48 | 51.56 | 0.14 |
| 24 | AME | Sal | 24.41 | 14.02 | 19.79 | 33.81 | 0.17 |
| 25 | AME | Sal | 19.51 | 22.46 | 22.95 | 45.41 | 0.01 |
| 26 | AME | Sal | 23.04 | 38.43 | 34.86 | 73.29 | -0.05 |
| 27 | AME | Sal | 10.70 | 16.69 | 58.52 | 75.21 | 0.56 |
| 28 | AME | Sal | 33.29 | 30.11 | 39.50 | 69.61 | 0.13 |
| 29 | AME | Sal | 19.68 | 19.01 | 29.42 | 48.43 | 0.21 |
| 1 | Sed | ANI | 19.16 | 25.95 | 28.74 | 54.69 | 0.05 |
| 2 | Sed | ANI | 19.42 | 46.44 | 36.86 | 83.30 | -0.12 |
| 3 | Sed | ANI | 20.02 | 17.34 | 37.92 | 55.26 | 0.37 |
| 4 | Sed | ANI | 3.89 | 3.82 | 2.01 | 5.83 | -0.31 |
| 5 | Sed | ANI | 17.14 | 12.87 | 14.21 | 27.08 | 0.05 |
| 6 | Sed | ANI | 25.08 | 28.82 | 37.18 | 66.00 | 0.13 |
| 7 | Sed | ANI | 23.29 | 30.23 | 28.75 | 58.98 | -0.03 |
| 8 | Sed | ANI | 25.27 | 40.79 | 19.18 | 59.97 | -0.36 |
| 9 | Sed | ANI | 11.41 | 22.12 | 14.54 | 36.66 | -0.21 |
| 10 | Sed | ANI | 23.46 | 21.45 | 24.83 | 46.28 | 0.07 |
| 11 | Sed | ANI | 17.56 | 14.01 | 12.16 | 26.17 | -0.07 |
| 12 | Sed | ANI | 19.18 | 21.77 | 41.70 | 63.47 | 0.31 |
| 13 | Sed | ANI | 24.10 | 30.40 | 22.83 | 53.23 | -0.14 |
| 14 | Sed | ANI | 30.83 | 20.42 | 28.64 | 49.06 | 0.17 |
| 15 | Sed | ANI | 37.58 | 24.42 | 17.19 | 41.61 | -0.17 |
| 16 | AME | ANI | 21.69 | 32.17 | 31.39 | 63.56 | -0.01 |
| 17 | AME | ANI | 26.89 | 14.20 | 13.69 | 27.89 | -0.02 |
| 18 | AME | ANI | 7.23 | 3.25 | 13.91 | 17.16 | 0.62 |
| 19 | AME | ANI | 22.52 | 24.33 | 11.41 | 35.74 | -0.36 |
| 20 | AME | ANI | 19.98 | 22.58 | 30.87 | 53.45 | 0.16 |
| 21 | AME | ANI | 5.38 | 4.74 | 6.14 | 10.88 | 0.13 |
| 22 | AME | ANI | 22.02 | 33.26 | 32.79 | 66.05 | -0.01 |
| 23 | AME | ANI | 27.69 | 38.43 | 36.42 | 74.85 | -0.03 |
| 24 | AME | ANI | 17.34 | 8.69 | 5.74 | 14.43 | -0.20 |
| 25 | AME | ANI | 11.48 | 10.42 | 4.94 | 15.36 | -0.36 |
| 26 | AME | ANI | 35.75 | 25.11 | 43.93 | 69.04 | 0.27 |
| 27 | AME | ANI | 5.36 | 17.37 | 10.06 | 27.43 | -0.27 |
| 28 | AME | ANI | 34.80 | 22.08 | 46.51 | 68.59 | 0.36 |
| 29 | AME | ANI | 18.44 | 25.82 | 23.95 | 49.77 | -0.04 |

Sed: sedentary control; AME: acute moderate exercise; Sal: saline; ANI: anisomycin; TDM: total distance moved; ET-F: exploration time of familiar (F) location object; ET-N: exploration time of familiar (N) location object; TE: total (F+N) object exploration time; DR: discrimination ratio.
